# Impact of Antenatal Corticosteroids on Brain Function and Underlying Mechanisms in Preclinical Studies: Protocol for a Systematic Review

**DOI:** 10.2196/84522

**Published:** 2026-03-27

**Authors:** Mauritz F Herselman, Tara Hislop, Sebastian D McBride, Mitchell C Lock, Janna L Morrison, Kathryn L Gatford

**Affiliations:** 1 School of Pharmacy and Biomedical Science, University of Adelaide, Adelaide Health & Medical Sciences building, corner George St and North Terrace, Adelaide, SA, 5005, Australia, +61 883134158; 2 Robinson Research Institute, College of Health, University of Adelaide, Adelaide, SA, Australia; 3 Department of Life Sciences, Aberystwyth University, Aberyswyth, United Kingdom

**Keywords:** corticosteroid, glucocorticoids, pregnancy, brain, pituitary-adrenal system, gestation, neurodevelopment, animal models

## Abstract

**Background:**

Antenatal corticosteroid (ACS) treatment matures the fetal lung and reduces risks of neonatal morbidity and mortality in babies born preterm. However, ACS treatment also impacts the brain and stress regulatory systems, with increasing clinical evidence for adverse long-term impacts. Preclinical studies are important to investigate the mechanisms for these impacts.

**Objective:**

This systematic review aims to synthesize the best available evidence describing how in utero exposure to ACS affects brain function, the underlying mechanisms, and the hypothalamic-pituitary-adrenal axis in nonhuman mammalian species.

**Methods:**

This review will include peer-reviewed, primary studies that report measures of brain function (eg, learning, behavior) and the underlying mechanisms (eg, brain size, neuron number, myelination, hypothalamic-pituitary-adrenal axis function) in nonhuman mammals exposed in utero to ACS in the second half of pregnancy, in comparison to unexposed individuals. Initial search terms include (corticosteroid* OR glucocorticoid*) AND (antenatal OR fetal* OR pregnan*) AND (brain OR neurodevelopment*). We searched PubMed, Embase, MEDLINE (Ovid), Web of Science, Scopus, and ProQuest for English language publications without date restrictions. Two independent reviewers will perform abstract screening, data extraction, and quality assessment. Antenatal corticosteroid treatment information, study design, methods, and outcomes will be reported for each study.

**Results:**

A narrative synthesis will be presented following standard guidelines. A dose-response meta-analysis will be performed where at least three studies report the same outcome following in utero exposure to the same steroid. As of February 21, 2026, title and abstract screening were completed for 42,024 of 56,493 records, with 40,795 records excluded. Searches will be updated in June-July 2026 to include sources published to the end of 2025. Publication is planned for 2027.

**Conclusions:**

This review will inform future research including intervention studies to reduce the adverse effects of antenatal corticosteroids on the brain.

## Introduction

Antenatal treatment with corticosteroids (ACS)—discovered in sheep [1], translated rapidly to clinical trials [2], and now standard care for women at risk of preterm delivery [3]—is the only effective antenatal intervention to date for preventing neonatal respiratory distress and improving survival of preterm babies. Maternally delivered ACS rapidly cross the placenta to the fetal circulation, where they activate the glucocorticoid receptor (GR) in fetal lungs to promote lung maturation, thereby reducing the risk of respiratory distress syndrome by 29% and neonatal death by 22% [3]. The two most commonly used synthetic corticosteroids, dexamethasone and betamethasone, have similar efficacy in preventing respiratory distress syndrome or death of offspring when given to women at risk of preterm birth [4]. However, ACS treatment lacks specificity, resulting in off-target effects, particularly to the developing brain, which also expresses the GR [5-7] and is equally exposed to ACS in the fetal circulation. Clinical concerns center on growing evidence for adverse neurodevelopmental effects of ACS, including greater stress responses and behavioral problems [8,9]. In the largest childhood follow-up to date, mental and behavioral disorders occurred in 12.01% of ACS-exposed individuals compared with 6.45% of nonexposed individuals within the entire cohort of preterm and term-born children [10]. However, as recently reviewed, there is evidence that the effects of ACS exposure vary with gestational age at birth, with benefit in those born preterm and evidence of harm mostly in those born late preterm or at term [11]. For example, exposure to a single course of ACS versus nonexposure was associated with lower odds of neurodevelopmental impairment at 18‐22 months of age in infants born preterm (adjusted odds ratio 0.69, 95% CI 0.57‐0.84, low certainty of evidence) [12]. A recent systematic review of outcomes in children born at term identified that early exposure to ACS was associated with greater risks of adverse short-term (neonatal intensive care admission, reduced head circumference, intubation) and long-term outcomes (diagnosis of neurodevelopmental or behavioral disorder), albeit with low or very low certainty, reflecting limited availability of evidence [13]. This group is important, because 40% of ACS-treated mothers do not deliver preterm [13], putting their babies at risk of side effects without respiratory benefit. It is also not clear whether outcomes differ between steroid types, despite no difference in risks of neurosensory disability between children exposed to dexamethasone or betamethasone before birth [14]. Clinical evidence for the mechanistic basis of ACS-mediated changes in neurodevelopment is limited. Although there is moderate-quality evidence that ACS exposure reduces the risk of intraventricular hemorrhage [3], there is limited clinical data on the impacts of ACS exposure on structural or molecular determinants of brain function. Many, but not all, studies identified in a recent scoping review reported reduced head circumference in newborns and children who were exposed to ACS but born late preterm or at term [15]. In two small magnetic resonance imaging studies within this analysis of outcomes in late preterm and term-born offspring, the ACS-exposed group also had a thinner cortex at 6‐10 years of age and indications of reduced brain gyrification at birth, including lower surface area and cortex convolution index [15]. These structural deficits likely occur along with reprogramming of the hypothalamic-pituitary-adrenal (HPA) axis since exogenous corticosteroids can interfere with negative feedback of this system [16,17]. Indeed, the HPA axis is suppressed in neonates after ACS exposure, which may persist for several months after birth [18]. Longer-term reprogramming of the HPA axis involves alterations in GR expression in the hippocampus and hypothalamus, which may exacerbate stress reactivity and contribute to behavioral disorders in children and adults [17]. For example, ACS exposure increased cortisol responses to psychosocial stress in 6‐11 year olds born at term, particularly in girls [19].

Preclinically, ACS induces impaired cognition and altered stress responses, as in humans. Neurobehavioral assessments in rodents identified lower activity in open field tests, indications of increased anxiety-like behavior in the elevated plus maze test, more time immobile in forced swim tests, and increased startle responses in those exposed to ACS [20]. Functional impacts of ACS have also been observed in primates, evaluated using the Cambridge Neuropsychological Test Automated Battery system in term-born male and female baboons exposed to repeat courses of clinical doses of betamethasone, compared to offspring of saline-treated mothers [21]. In this study, performance on the moving stimulus task used during touchscreen training, as well as in a simple reversal task, was poorer in betamethasone than control offspring, but only in females [21]. Mechanistically, neurological changes that might underlie altered behavior and cognition after ACS include greater GR expression and lower neuron densities, particularly serotonergic and dopaminergic neurons, with most studies conducted in rodents exposed to high concentrations of dexamethasone [20]. ACS exposure also induces immune and vascular changes in the fetal brain. For example, in preterm-born lambs delivered at gestational day 125 (term ~150 days), exposure to betamethasone was associated with greater cerebral white matter inflammation and vascular permeability [22].

In contrast to the multiple systematic reviews of neurodevelopmental impacts of in utero exposure to ACS in humans, the systematic or scoping reviews describing these outcomes in nonhuman species are of narrower scope and do not fully reflect the clinical context. These include systematic reviews of the impacts of repeat corticosteroid exposure [23], of long-term impacts of ACS, excluding brain changes observed before and shortly after birth [20], and a more recent review of ACS in preclinical models of fetal growth restriction [24]. We therefore plan to conduct a comprehensive and contemporary systematic evaluation of the evidence for impacts of in utero exposure to ACS on the brains of nonhuman mammals. Due to the depth and range of evidence, we will synthesize our results into three reviews, one describing the impacts of ACS on brain function, another discussing the impacts of ACS on underlying mechanisms that may contribute to altered function, and the third describing the impacts of ACS on the HPA axis, including basal and stress-induced function.

## Methods

### Review Question

What are the impacts of in utero ACS administration on brain function, the underlying mechanisms, and the HPA axis in nonhuman mammalian species?

### Search Strategy

An initial search of PubMed and PROSPERO (International Prospective Register of Systematic Reviews) was undertaken to identify articles on the topic and to assess knowledge gaps. A full search strategy was developed using indexed terms and text words within the titles and abstract (Table 1). This search string was then modified to include syntax specific to each individual database. This search string was validated by checking for the inclusion of a set of 11 key references reporting outcomes spanning brain function, determinants, and HPA axis measures in the output of each search [25-35]. We searched the following electronic databases for published evidence: PubMed, Embase, MEDLINE (Ovid), Web of Science, Scopus, and ProQuest. Searches will be updated in June-July 2026 to identify and include all sources published or accepted for publication to the end of 2025. Publication is planned for 2027.

**Table 1.** Search strategy.

Database	Search string
MEDLINE (PubMed)	(Corticosteroid* OR Glucocorticoid* OR Glucocorticoids [mh] OR Betamethasone OR Betamethasone [mh] OR Dexamethasone OR Dexamethasone [mh] OR Cortisol* OR Hydrocortisone* OR Hydrocortisone [mh] OR Corticosterone* OR Corticosterone [mh]) AND (Brain* OR Brain [mh] OR “Brain development” OR Neurodevelopment* OR Neuron* OR Neurons [mh] OR Mental Disorders [mh] OR Intelligence OR Learning [mh] OR Memory [mh] OR Cogniti* OR Cognition [mh] OR Behaviour* OR Behavior* OR Psycholog* OR Anxiety OR Depress* OR Stress* OR “hypothalamo-pituitary-adrenal axis” OR “HPA axis” OR Adrenal glands [mh] OR Adrenocorticotropic hormone [mh] OR Corticotropin-releasing hormone [mh] OR Cortisol OR Corticosterone* OR Corticosterone [mh] OR Hippocamp* OR Hippocampus [mh] OR “Corpus Callosum” OR Corpus Callosum [mh] OR Myelin* OR Neuroglia[mh] OR Oligodendrocyte* OR White matter [mh] OR Gray matter [mh] OR Cerebrum [mh] OR Cerebell* OR Neuroanatom* OR Amygdala* OR Astrocyte*) AND (Antenatal* OR Prenatal* OR Pregnancy Complications [mh] OR Preterm OR Fetus* OR Fetus [mh] OR Foetus* OR Fetal* OR Foetal* OR “Embryonic and Fetal Development” [mh] OR Gestat* OR Pregnan* OR Pregnancy [mh])
MEDLINE (Ovid)	(Corticosteroid*.mp. OR exp Glucocorticoids/ OR Glucocorticoid*.mp. OR Betamethasone.mp. OR exp Betamethasone/ OR Dexamethasone.mp. OR exp Dexamethasone/ OR Cortisol*.mp. OR Hydrocortisone*.mp. OR exp Hydrocortisone/ OR Corticosterone*.mp. OR exp Corticosterone/) AND (Brain*.mp. OR exp Brain/ OR “Brain development”.mp. OR Neurodevelopment*.mp. OR Neuron*.mp. OR exp Neurons/ OR exp Mental Disorders/ OR Intelligence.mp. OR exp Learning/ OR exp Memory/ OR Cogniti*.mp. OR exp Cognition/ OR Behavio$r*.mp. OR Psycholog*.mp. OR Anxiety.mp. OR Depress*.mp. OR Stress*.mp. OR hypothalamo-pituitary-adrenal axis.mp. OR HPA axis.mp. OR exp Adrenal glands/ OR exp Adrenocorticotropic hormone/ OR exp Corticotropin-releasing hormone/ OR Cortisol.mp. OR Corticosterone*.mp. OR exp Corticosterone/ OR Hippocamp*.mp. OR exp Hippocampus/ OR Corpus Callosum.mp. OR exp Corpus Callosum/ OR Myelin*.mp. OR exp Neuroglia/ OR Oligodendrocyte*.mp. OR exp White matter/ OR exp Gray matter/ OR exp Cerebrum/ OR Cerebell*.mp. OR Neuroanatom*.mp. OR Amygdala*.mp. OR Astrocyte*.mp.) AND (Antenatal*.mp. OR exp Pregnancy Complications/ OR Prenatal*.mp. OR Preterm.mp. OR F$etus*.mp. OR exp Fetus/ OR F$etal*.mp. OR exp “Embryonic and Fetal Development”/ OR Gestat*.mp. OR Pregnan*.mp. OR exp Pregnancy/)
ProQuest Dissertations & Theses Global	((TI,AB,DISKW(Steroid* OR Corticosteroid* OR cortisol* OR Betamethasone OR Dexamethasone OR Glucocorticoid* OR Corticosterone* OR Hydrocortisone) OR SU(Corticosteroids OR Steroids)) AND (TI,AB,DISKW(Brain OR Neurodevelopment* OR Neuron* OR “Neurological disorder*” OR “Mental disorder*” OR Intelligence OR Learning OR Memory OR Cogniti* OR Recognition OR Behavio$r* OR Psycholog* OR Anxiety OR Depress* OR “Mood disorders” OR Stress* OR “HPA axis” OR “hypothalamo-pituitary-adrenal axis” OR Hypothalamus OR “Adrenal gland*” OR Adrenocorticotropi* OR Corticotropin OR “Corticotropin-releasing” OR Cortisol OR Corticosterone OR Hippocamp* OR “Corpus Callosum” OR Myelin* OR Neuroglia* OR Glia* OR Oligodendro* OR “White matter” OR “Gr?y matter” OR Cerebr* OR Cerebell* OR Neuroanatom* OR Amygdala* OR Astrocyt* OR Neurotransm*) OR SU(“Brain development” OR “Neurological disorders” OR Neurotoxicity OR Neurosciences OR Neurons OR Learning OR Memory OR “Cognition & reasoning” OR Neurotransmitters OR Behavior OR “Psychosocial development” OR “Mood disorders” OR Anxiety)) AND (TI,AB,DISKW(Prenatal* OR Antenatal* OR Pregnan* OR Preterm OR F$etal* OR F$etus* OR Gestat*) OR SU(“Prenatal care” OR “Prenatal development” OR Fetuses))) AND la.exact(“ENG”)
Embase	(exp Corticosteroid/ or exp *Betamethasone/ or exp *Dexamethasone/ or exp *Hydrocortisone/ or exp *Corticosterone/ or (Corticosteroid* or cortisol* or Betamethasone or Dexamethasone or Glucocorticoid* or Corticosterone* or Hydrocortisone).ti,ab,kf,tn.) and (Brain*.ti,ab,kf. or exp *Brain/ or "Brain development".ti,ab,kf. or Neurodevelopment*.ti,ab,kf. or Neuron*.ti,ab,kf. or exp *Nerve cell/ or exp *Mental disease/ or Intelligence.ti,ab,kf. or exp *Learning/ or exp *Memory/ or Cogniti*.ti,ab,kf. or exp *Cognition/ or Behavio$r*.ti,ab,kf. or Psycholog*.ti,ab,kf. or Anxiety.ti,ab,kf. or Depress*.ti,ab,kf. or Stress*.ti,ab,kf. or "hypothalamo-pituitary-adrenal axis".ti,ab,kf. or "HPA axis".ti,ab,kf. or exp *Adrenal gland/ or exp *Corticotropin/ or Cortisol.ti,ab,kf. or Corticosterone*.ti,ab,kf. or exp *Corticosterone/ or Hippocamp*.ti,ab,kf. or exp *Hippocampus/ or "Corpus Callosum".ti,ab,kf. or exp *Corpus Callosum/ or Myelin*.ti,ab,kf. or exp *Myelin/ or exp *Glia cell/ or Oligodendrocyte*.ti,ab,kf. or "White matter".ti,ab,kf. or "Gray matter".ti,ab,kf. or Cerebell*.ti,ab,kf. or Neuroanatom*.ti,ab,kf. or Amygdala*.ti,ab,kf. or Astrocyte*.ti,ab,kf.) and (Antenatal*.ti,ab,kf. or exp *Pregnancy complication/ or Prenatal*.ti,ab,kf. or exp *Prenatal growth/ or Preterm.ti,ab,kf. or F$etus*.ti,ab,kf. or exp *Fetus/ or exp *Fetus growth/ or F$etal*.ti,ab,kf. or exp *Prenatal development/ or Gestat*.ti,ab,kf. or Pregnan*.ti,ab,kf. or exp *Pregnancy/)
Web of Science	(Corticosteroid* OR cortisol* OR Betamethasone OR Dexamethasone OR Glucocorticoid* OR Corticosterone* OR Hydrocortisone) AND (Brain* OR “Brain development” OR Neurodevelopment* OR Neuron* OR “Mental Disorders” OR Intelligence OR Learning OR Memory OR Cogniti* OR Behavio$r* OR Psycholog* OR Anxiety OR Depress* OR Stress* OR “hypothalamo-pituitary-adrenal axis” OR “HPA axis” OR Adrenal* OR “Adrenocorticotropic hormone” OR “Corticotropin-releasing hormone” OR Cortisol OR Corticosterone* OR Hippocamp* OR “Corpus Callosum” OR Myelin* OR Neuroglia* OR Oligodendrocyte* OR “White matter” OR “Gr?y matter” OR Cerebr* OR Cerebell* OR Neuroanatom* OR Amygdala* OR Astrocyte*) AND (Antenatal* OR Prenatal* OR Preterm OR F$etus* OR F$etal* OR Gestat* OR Pregnan*)
Scopus	TITLE-ABS-KEY ((Corticosteroid* OR cortisol* OR Betamethasone OR Dexamethasone OR Glucocorticoid* OR Corticosterone* OR Hydrocortisone) AND (Brain* OR “Brain development” OR Neurodevelopment* OR Neuron* OR “Mental Disorders” OR Intelligence OR Learning OR Memory OR Cogniti* OR Behavior* OR Psycholog* OR Anxiety OR Depress* OR Stress* OR “hypothalamo-pituitary-adrenal axis” OR “HPA axis” OR Adrenal* OR “Adrenocorticotropic hormone” OR “Corticotropin-releasing hormone” OR Cortisol OR Corticosterone* OR Hippocamp* OR “Corpus Callosum” OR Myelin* OR Neuroglia* OR Oligodendrocyte* OR “White matter” OR “Gr?y matter” OR Cerebr* OR Cerebell* OR Neuroanatom* OR Amygdala* OR Astrocyte*) AND (Antenatal* OR Prenatal* OR Preterm OR Fetus* OR Fetal* OR Gestat* OR Pregnan*))

### Study Selection

Database search results will be imported to Covidence (Covidence systematic review software, Veritas Health Innovation), where reviewers can collaboratively screen sources for inclusion. Two assessors (any two of the review authors) will independently review sources using eligibility criteria (Table 2) at title, abstract, and full-text levels. Assessors will be blinded during screening. Uncertainties or conflicting decisions at each stage will be resolved through discussion and consensus, with at least one other member of the review team.

**Table 2.** Eligibility criteria for abstract screening.

	Inclusion criteria	Exclusion criteria
Population	Nonhuman mammals Healthy pregnancies and offspring	Humans Nonmammalian species Genetically modified strains Unhealthy pregnancies and offspring or models exposed to additional experimental manipulations
Exposure of interest	In utero exposure to exogenous corticosteroids during the second half of gestation Specific corticosteroids: dexamethasone, cortisol, corticosterone, or hydroxycortisol with no restrictions on the corticosteroid formulation, dose, or route of administration	Corticosteroid administration not continuing after 0.5 gestation Administration of postnatal steroids Manipulations of endogenous steroids (eg, maternal restraint stress)
Comparator	Concurrently studied placebo or untreated comparison group assessed at the same fetal or postnatal age	Studies lacking a control group evaluated at the same fetal or postnatal age
Outcomes	Functional brain outcomes including measures of cognition, intelligence, learning, memory, attention, social and other behavior, psychological disorders, and executive function Structural determinants of brain function including gross measures of brain size and shape such as cortical folding, regional volumes, and brain pathology; measures of brain connectivity; measures of numbers, density, proportions, proliferation, and apoptosis for cell types within the brain such as neurons, glia, and astrocytes; measures of cellular morphology such as pruning, dendrites, axons, branching, volumes, and myelination; synaptic neurotransmitter abundance and receptors; measures of blood-brain barrier function, oxidative stress, and inflammation Molecular determinants of brain function including gene and protein expression in pathways related to glucocorticoid signaling, neurodevelopment, myelination, apoptosis/proliferation, neurotransmitters, and oxidative stress, and epigenetic measures relating to determinants of brain function Basal and stress-induced measures of hypothalamic-pituitary-adrenal axis function including circulating adrenocorticotropin, cortisol-releasing hormone, and corticosteroid concentrations Structural and molecular determinants of hypothalamic-pituitary-adrenal axis function including adrenal size, glucocorticoid receptor, and steroidogenic enzyme expression	No relevant outcomes
Types of studies	Primary (original) studies Peer-reviewed literature (published or in press, including theses) Available in English	Conference abstracts, reviews, editorials, trial protocols Literature that has not undergone peer review (eg, preprints) Full text not available in English

### Data Extraction

Data extraction will be performed by two independent reviewers using author-designed fields in Covidence that will reflect the standardized JBI data extraction [36]. Uncertainties or conflicting decisions at each stage will be resolved through discussion and consensus with at least one other member of the review team. Study authors will be contacted to request any missing or additional data. The extracted data will include citation information, species, strain, and normal term gestation of experimental animals used and information regarding study design including sample size (including number of mothers and number of offspring), method of allocation to treatments, timing of delivery, and assessment blinding. Extracted data relating to the ACS exposure will include the type of corticosteroid used, the dose including the formulation and route of administration, and the timing and duration of administration expressed in days of gestation and as a fraction of term. Extracted data related to outcomes will include sample size, sex, and postnatal age at assessment, details of the functional outcomes or brain regions analyzed, and information regarding the methodology used to measure outcomes. Where possible, the means, standard deviations, and percentage differences will be extracted for each treatment group and outcome. Where outcomes are only presented in figures, the numeric data (means and variance) will be extracted using WebPlotDigitizer v5 (Automeris LLC). Where further data or clarification are required for a study, the authors will be contacted via email. A minimum of three attempts at monthly intervals will be made to contact authors. Following extraction, we will cross-reference author names, experimental design, and study dates to identify multiple publications originating from the same experiment to prevent redundant data extractions arising from multiple publications. Where studies report overlapping data, such as multiple reports of the same outcome from an experimental cohort, only data from the largest study or more relevant exposures will be included. Where the same outcomes are reported in identical cohorts, the earliest publication will be retained. In studies where multiple treatment groups are compared against a single shared control group, the control group sample size will be divided equally across the comparison arms.

### Assessment of Methodological Quality

Methodological quality will be assessed through the use of the Systematic Review Center for Laboratory Animal Experimentation Risk of Bias (SYRCLE RoB) tool [37]. Critical appraisal will be conducted by two independent reviewers, and judgment disagreements will be resolved through discussion and consensus or a third review if necessary. Appraisal results will be reported in table format.

### Data Synthesis

A narrative synthesis will be presented following standard guidelines. A dose-response meta-analysis will be performed where at least 3 studies report the same outcome following in utero exposure to the same corticosteroid with comparable timing and data are made available by authors. The litter will be treated as the primary unit of analysis. For studies that report data from multiple offspring per litter without adjustment, the litter mean will be used as a single data point for each measured outcome. To ensure that these dose-response analyses are biologically meaningful across different mammalian species, doses will be standardized using allometric scaling (based on body surface area) to calculate human-equivalent dosing where possible. Where sufficient data are available, subgroup analyses will be performed within different species and biological sexes. Studies with missing data where authors do not respond to data requests will be included in the narrative synthesis but will be excluded from meta-analyses. Studies that report mean values for groups without measures of variance will be excluded from the meta-analysis but will be included in the narrative synthesis. For instance, where studies in the functional review report overlapping cohorts or outcomes with the underlying mechanisms and stress response reviews, we will cross-reference these findings to highlight consistent patterns of association across the different domains.

### Assessing Certainty in the Findings

For any outcomes where meta-analysis is able to be performed, the Grading of Recommendations, Assessment, Development and Evaluation (GRADE) approach will be used to grade the certainty of evidence [38]. Following this, a Summary of Findings will be created using GRADEpro GDT (McMaster University). Independent, blinded assessment of the certainty of evidence will be carried out by two reviewers, with any potential disagreements resolved through discussion or the involvement of a third reviewer. The Summary of Findings will present, where appropriate, mean differences, a ranking of the quality of the evidence based on the risk of bias, heterogeneity, precision, and risk of publication bias of the review results.

### Dissemination Plan

The results of this systematic review will be disseminated through peer-reviewed publications.

## Results

**Figure 1.**
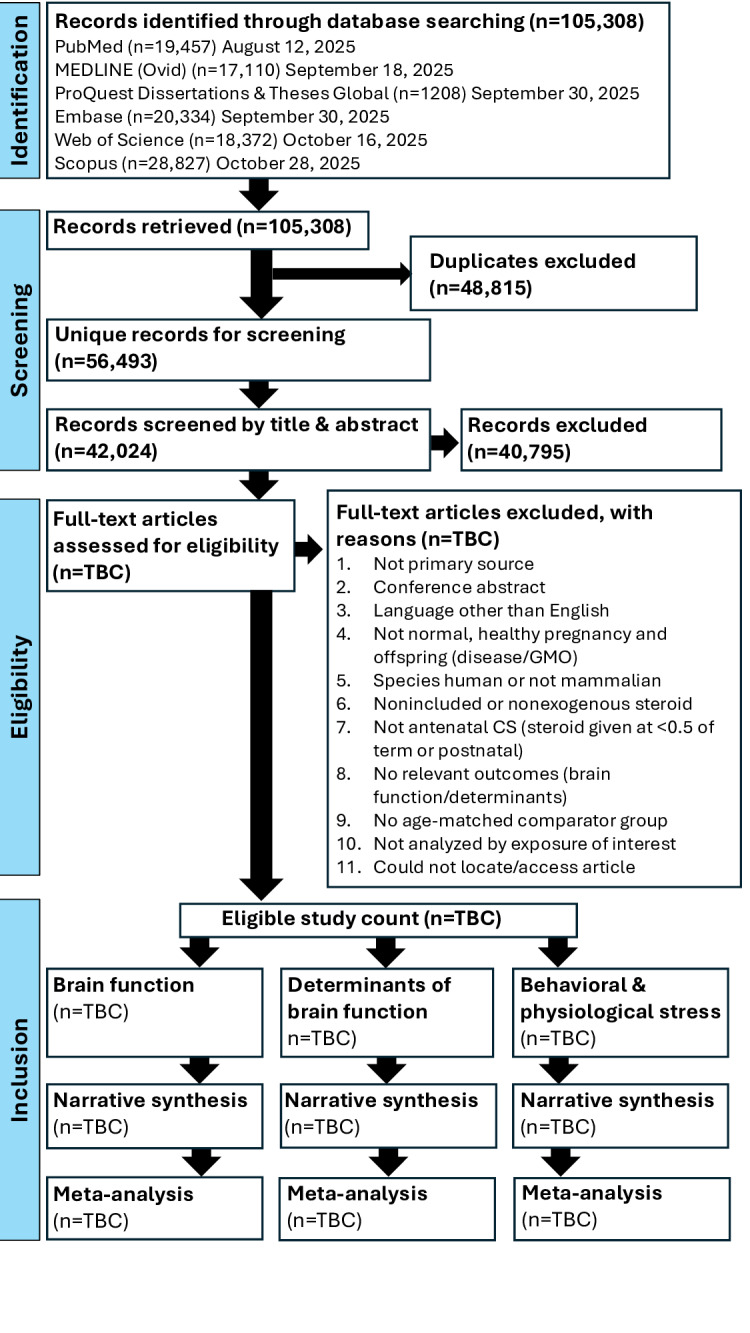
PRIMSA (Preferred Reporting Items for Systematic Reviews and Meta-Analyses) flow diagram of study selection as of February 21, 2026. CS: corticosteroid; GMO: genetically modified organisms; TBC: to be confirmed.

Initial searches were performed between August 12, 2025, and October 28, 2025, yielding 105,457 records. Search results from each database were downloaded to EndNote, duplicates and retractions were removed, and the remaining 105,308 sources were uploaded into Covidence (Figure 1). Records retrieved from more than one database were removed as duplicates by Covidence (n=48,698) or manually removed as duplicates during screening (n=117), resulting in 56,493 unique records (Figure 1). As of February 21, 2026, a total of 42,024 records have undergone title and abstract screening, with 40,795 records excluded (Figure 1). Publication is planned for 2027.

## Discussion

### Principal Findings

We hypothesize that this systematic review will reveal a dose-dependent relationship between ACS exposure and alterations in neurodevelopmental outcomes. Specifically, ACS exposure may be associated with increased anxiety-like behaviors and deficits in spatial learning and memory across various mammalian species. We expect to find evidence of reduced neuronal density and altered myelination patterns in brain regions relevant to behavior and altered HPA axis sensitivity. We hypothesize that the impacts of ACS will differ by biological sex, potentially suggestive of greater vulnerability in one sex regarding cognitive tasks or stress-reactivity. Finally, we anticipate that, while basic mechanisms of glucocorticoid action are conserved across species, the timing of exposure relative to brain maturation may contribute to differences in impacts in different species.

### Comparison to Prior Work

Previous scoping and systematic reviews of the effects of ACS on neurodevelopment and stress responses have primarily focused on clinical outcomes in human cohorts [12,15,18]. The systematic or scoping reviews describing these outcomes in nonhuman species are of narrower scope, do not fully reflect the clinical context, and/or require updating [20,23,24]. Unlike clinical studies that may limited by confounding environmental variables, synthesis of preclinical evidence allows for a more controlled evaluation of dose-response relationships and tissue-specific molecular changes.

### Limitations

This systematic review will have several limitations. Given that we are excluding publications not in English at screening, there is a possibility that we will miss relevant studies published in other languages, although these will be listed in the exclusion reasons for transparency [39]. It is not clear whether meta-analyses of outcomes will be possible given the heterogeneity of timings of steroid administration and outcome evaluation and the variety of species, antenatal steroid regimens, and outcome assessments evident in previous reviews [20]. Effect sizes may also be overestimated due to the potential nonpublication of null results in preclinical research [40].

## Abbreviations

ACS: antenatal corticosteroids
GR: glucocorticoid receptor
GRADE: Grading of Recommendations, Assessment, Development and Evaluation
HPA: hypothalamic-pituitary-adrenal
SYRCLE RoB: Systematic Review Centre for Laboratory Animal Experimentation Risk of Bias
